# Effect of Trimetazidine in Patients Undergoing Percutaneous Coronary Intervention: A Meta-Analysis

**DOI:** 10.1371/journal.pone.0137775

**Published:** 2015-09-14

**Authors:** Ying Zhang, Xiao-juan Ma, Da-zhuo Shi

**Affiliations:** 1 Graduate School, Beijing University of Chinese Medicine, Beijing, China; 2 China Heart Institute of Chinese Medicine, China Academy of Chinese Medical Sciences, Beijing, China; 3 Cardiovascular Diseases Center, Xiyuan Hospital, China Academy of Chinese Medical Sciences, Beijing, China; Shenzhen Institutes of Advanced Technology, CHINA

## Abstract

Optimizing the metabolism of the myocardium is a new strategy for patients with ischemic heart disease. Many studies have reported beneficial effects of trimetazidine (TMZ) on the clinical prognosis of patients with ischemic heart disease, but whether these beneficial effects are extended to patients undergoing percutaneous coronary intervention (PCI) remains uncertain. A meta-analysis was performed to evaluate the effect of TMZ on patients undergoing PCI. We conducted an electronic search of PubMed, Cochrane databases, the China National Knowledge Infrastructure, and Chinese Biological Medicine Database to identify randomized controlled trials. Methodological quality was assessed according to the Jadad scale score, and the meta-analysis was performed using Cochrane Collaboration RevMan 5.2 and Comprehensive Meta-Analysis. Dichotomous data were analyzed using relative risk (RR) or odds ratio (OR) with effect size indicated by the 95% confidence interval (CI), and continuous variables were analyzed using weighted mean differences (WMD) with effect size indicated by the 95% CI. Sensitivity analysis was performed by changing the statistical methods and effect model. Nine studies involving a total of 778 patients were included in this meta-analysis. Additional use of TMZ significantly improved the left ventricular ejection fraction (WMD: 3.11, 95% CI: [2.26, 3.96]) and reduced elevated cardiac troponin Ic level (RR: 0.69, 95% CI: [0.48, 0.99]), angina attacks during PCI (OR: 0.16, 95% CI: [0.07, 0.38]), and ischemic ST-T changes on the echocardiogram during PCI (RR: 0.76, 95% CI: [0.59, 0.98]). However, no significant difference was observed in serum BNP level 30 days after PCI between the experimental and control group. Additional use of TMZ for patients undergoing PCI may reduce myocardial injury during the procedure and improve cardiac function.

## Introduction

Although great advances have been made in the therapeutic methods for cardiovascular diseases, acute coronary syndrome remains a major cause of morbidity and mortality worldwide [1]. Currently, percutaneous coronary intervention (PCI) is an important treatment strategy for the management of stenotic coronary artery disease. Along with major technical advancements in PCI, the incidence of major complications, such as acute myocardial infarction, emergency coronary artery bypass graft surgery, and even cardiac death during PCI, is limited [2,3]. However, the PCI operation may induce coronary spasm or endothelial cell injury, and the debris from atherosclerotic plaques or thrombi may cause coronary artery distal embolization, thereby leading to myocardial ischemia or myocardial injury. Minor peri/post-procedural myocardial injury or necrosis plays a crucial prognostic role after PCI [4].

Optimizing the metabolism of the myocardium is a new strategy for patients with stenotic coronary artery disease. Trimetazidine (TMZ), known as an anti-ischemic agent, exerts its effect by selectively inhibiting long-chain 3-ketoacyl-CoA thiolase and directly stimulating pyruvate dehydrogenase. This action typically has the effect of shifting cardiac energy metabolism from fatty acid oxidation to glucose oxidation, which can preserve the necessary ATP level in myocardial cells, promote a decline in intracellular acidosis, and protect cardiac myocytes from calcium overload [5]. Thus, myocardial injury caused by free radicals is reduced, and myocardial necrosis is minimal [6, 7].

In the past decade, quite a few RCTs investigating the effect of TMZ administration before and/or after PCI on myocardial injury and cardiac function have been reported. These studies demonstrated that TMZ exerted a beneficial effect on patients undergoing PCI [8, 9]; however, they all involved small sample sizes. Therefore, we conducted a meta-analysis with the aim to provide systematic evidence of TMZ in patients undergoing PCI.

## Methods

### Search strategy

An electronic literature search was performed by two authors (YZ and XM) in the following databases: PubMed (1989–December 2014), Cochrane databases (1993–December 2014), Chinese Biological Medicine Database (CBM, 1990–December 2014), China National Knowledge Infrastructure Database (CNKI, 1989–December 2014). No language restriction was applied. Search terms including “Trimetazidine,” “Vastarel,” “Idaptan,” “percutaneous coronary intervention,” “PCI,” “PTCA,” “myocardial injury,” “myocardial damage,” and “cardiac function” were used individually and combined.

### Selection criteria

Inclusion criteria for studies were as follows: (a) type of study, studies that claimed to be a RCT were included, regardless of blinding; (b) type of participants, all patients undergoing PCI, regardless of gender and age, were included; (c) type of intervention, TMZ as a co-intervention with conventional drugs for patients undergoing PCI in comparison with patients receiving conventional drugs; (d) types of outcome measures, studies reporting at least one of the following outcomes were considered eligible: markers of myocardial injury (i.e., cardiac troponin Ic [cTnI], creatine kinase-MB), cardiac function parameters (i.e., left ventricular ejection fraction [LVEF], left ventricular end systolic diameter [LVESD], left ventricular end-diastolic volume [LVEDV], and B-type natriuretic peptide [BNP]).

### Data extraction and quality assessment

Two of the investigators (YZ and XM) evaluated all studies and independently extracted relevant data from each study using a structured table. To resolve the dispute through consultation, if necessary, the third investigator (DS) was consulted. Details about the characteristics of patients and studies, medication administration, intervention strategies, and clinical outcomes were extracted. The Jadad scale score was employed to assess the quality of included studies, and a numerical score between 1 and 7 was assigned as a quality measure of study design [10].

### Statistical analysis

Dichotomous data were analyzed using relative risk (RR) or odds ratio (OR) with effect size indicated by the 95% CI, and continuous variables were analyzed using weighted mean differences (WMD) with effect size indicated by 95% CI. Sensitivity analysis was performed by changing the statistical methods and effect model. Meta-regression was conducted to test whether there was an interaction between baseline clinical features (age, gender, and multivessel disease) and rates of elevated cTnI >2 times the upper limit of normal. Statistical heterogeneity was measured using the I^2^ statistic. Fixed-effect models were applied if there was no significant heterogeneity across studies (I^2^ < 50%); otherwise, random-effect models were applied. All P values were 2-tailed, and the threshold for statistical significance was set at 0.05. Statistical analyses were performed using the Cochrane Collaboration (Rev Man 5.2, Copenhagen, Denmark) and Comprehensive Meta-Analysis (CMA; Biostat, Inc., USA). There is no registered protocol for the present meta-analysis.

## Results

### Study selection

**Fig 1:**
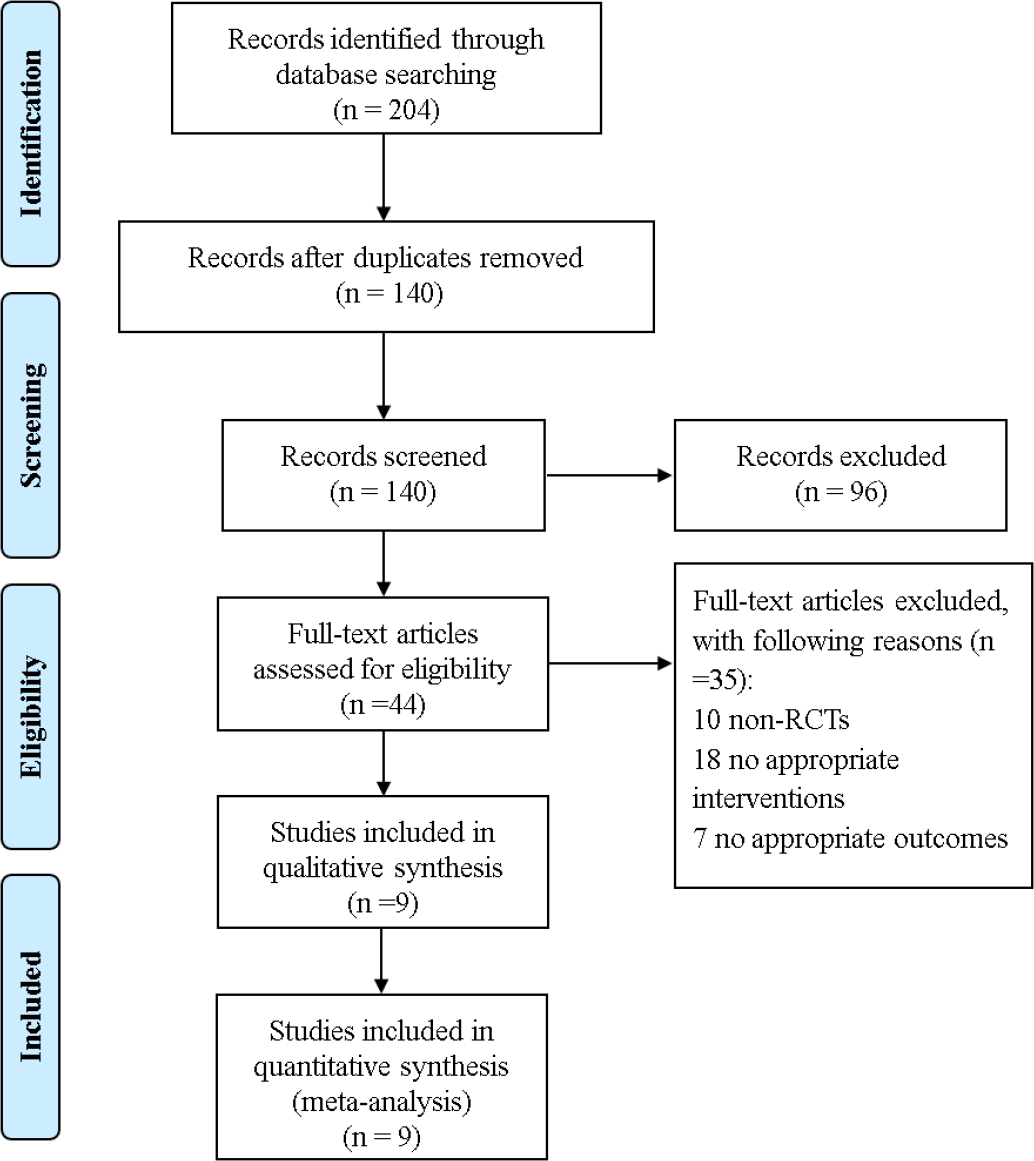
Flow Chart of Study Search and Selection.

Based on our inclusion criteria, 204 articles were retrieved according to the search strategy. Sixty-four trials were excluded after duplicates were removed. Through a preliminary review, 44 potential trials were identified, of which 35 were excluded for specific reasons listed in Fig 1. Only nine RCTs [2, 4, 11–17] met our inclusion criteria.

### Characteristics of studies

All nine included studies, with a total of 778 patients involved (396 in the experimental group and 382 in the control group), reported no significant difference in gender, age, and other basic information at baseline. Data concerning the characteristics of patients and studies, medication administration, intervention strategies, and clinical outcomes are shown in Tables 1 and 2. The results of the quality assessment are shown in Table 3.

**Table 1:** Description of the Characteristics of Trials Included in the Meta-Analysis.

Study	Individuals (E/C)	Age (E/C)(mean, y)	Male (E/C)	Multivessel disease n (%)	LVEF (E/C)(mean, %)	Intervention
**Demirelli et al. 2013**	22/23	57/59	20/15	0 (0)	58/57	PCI
**Birand et al. 1997**	26/25	49/53	-	30 (59)	54/55	PTCA
**Bonello et al. 2007**	136/130	68/53	98/92	0 (0)	65/62	PCI
**Polonski et al. 2002**	22/22	54/53	12/15	0 (0)	-	PTCA
**Chen et al. 2010**	54/47	63/63	41/36	-	63/62	PCI
**Labrou et al. 2007**	27/25	63/62	22/19	30 (58)	52/54	PCI
**Xu et al. 2013**	51/55	61/59	39/41	0 (0)	64/65	PCI
**Yu et al. 2012**	38/34	-	-	-	-	PCI
**Ma et al. 2010**	20/21	53/54	-	16 (39)	-	PCI

E = experimental group; C = control group; LVEF = left ventricular ejection fraction.

**Table 2:** TMZ Administration and Related Outcomes.

Study	TMZ	Outcomes
**Demirelli et al. 2013**	60 mg TMZ just prior to PCI and continued 60 mg daily (3×20 mg) for 1 mo after the procedure	Left ventricular function, BNP
**Birand et al. 1997**	60 mg daily (3×20 mg) TMZ after PTCA for 3 mo	LVEF
**Bonello et al. 2007**	60 mg TMZ 30 min prior to PCI	Ischemic ST-T changes on ECG during PCI
**Polonski et al. 2002**	60 mg TMZ daily at least 4 d before PTCA	cTnI, angina attacks during PCI
**Chen et al. 2010**	60 mg TMZ daily 5±2 d before PCI, 60 mg TMZ 30 min prior to PCI, 60 mg daily (3×20 mg) TMZ for 1 mo after PCI	LVEF, angina attacks during PCI, ischemic ST-T changes on ECG
**Labrou et al. 2007**	60 mg daily (3×20 mg) TMZ 15 d prior to PTCA and last 3 mo after PTCA	Left ventricular function
**Xu et al. 2013**	60 mg TMZ 0.5–1 h prior to PCI and continued 60 mg daily (3×20 mg) for 12 mo after the procedure	LVEF, cTnI
**Yu et al. 2012**	60 mg daily (3×20 mg) TMZ 7 d prior to PCI and 3 mo after PCI	LVEF, BNP
**Ma et al. 2010**	60 mg daily (3×20 mg) TMZ 3 d prior to PCI	cTnI

TMZ = trimetazidine; LVEF = left ventricular ejection fraction; ECG = electrocardiogram; PCI = percutaneous coronary intervention; PTCA = percutaneous transluminal coronary angioplasty; BNP = B-type natriuretic peptide; cTnI = troponin Ic.

**Table 3:** Quality Assessment of Included Studies.

Study	Generation of allocation sequence	Allocation concealment	Blindness	Withdrawal and drop out	Jadad score
**Demirelli et al. 2013**	1	1	1	1	4
**Birand et al. 1997**	1	1	1	1	4
**Bonello et al. 2007**	1	1	1	1	4
**Polonski et al. 2002**	1	1	1	1	4
**Chen et al. 2010**	2	1	1	1	5
**Labrou et al. 2007**	1	1	1	1	4
**Xu et al. 2013**	2	1	1	1	5
**Yu et al. 2012**	1	1	1	0	3
**Ma et al. 2010**	1	1	1	0	3

Jadad scale, points were determined as follows: I. generation of allocation sequence (computer-generated random numbers, 2 points; not described, 1 point; inappropriate method, 0 points); II. allocation concealment (central randomization, sealed envelopes or similar, 2 points; not described, 1 point; inappropriate or unused, 0 points); III. blindness (identical placebo tablets or similar, 2 points; inadequate or not described, 1 point; inappropriate or no double blinding, 0 points); IV. withdrawals and drop-outs (numbers and reasons are described, 1 point; not described, 0 points). The Jadad scale score ranges from 1 to 7; higher score indicates better RCT quality.

### Left ventricular function

**Fig 2:**
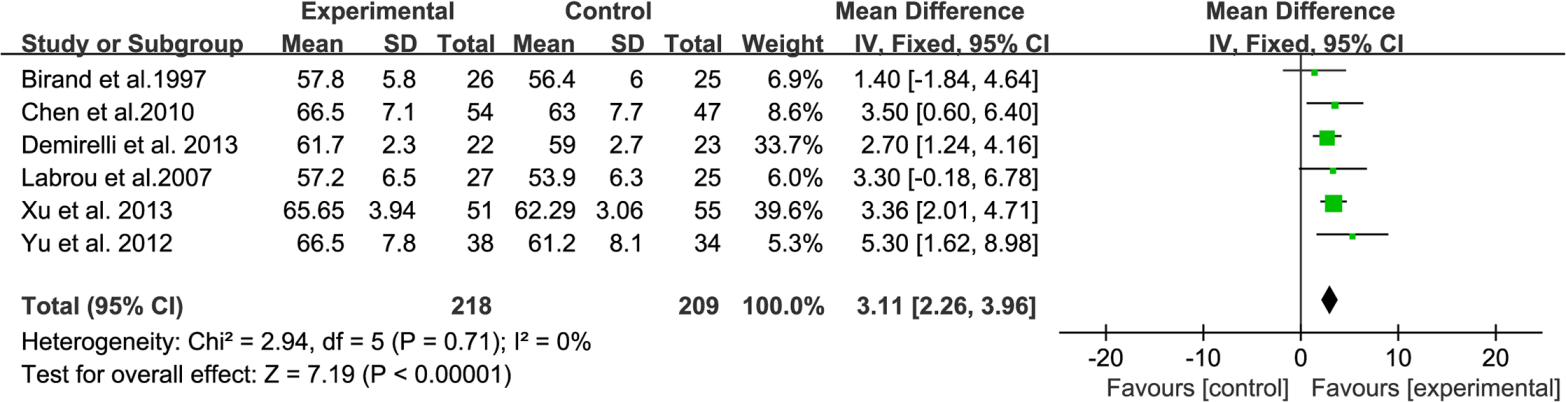
Forest Plots for LVEF.

Six of the included studies reported LVEF after follow-up, involving a total of 427 patients (218 in the experimental group and 209 in the control group). According to the test of heterogeneity (I^2^ = 0%, P = 0.71), a fixed-effects model was used to analyze the data. After pooling the data, the results of meta-analysis indicated that additional use of TMZ in the perioperative period of PCI was superior to conventional treatment in terms of LVEF (WMD: 3.11, 95% CI: [2.26, 3.96], P < 0.00001, Fig 2). Sensitivity analysis for timing of TMZ was performed by excluding the study in which TMZ was administered at post-operation [11]. The sensitivity analysis still showed positive results that were similar to the comprehensive results. (WMD: 3.24, 95% CI: [2.36, 4.12], P < 0.00001). One trial reported LVEDV as one of the outcomes. Compared with conventional medicine, additional use of TMZ reduced LVEDV significantly (71.9±7.7 cm^3^ from 75.1±13.1 cm^3^, P = 0.01). Another trial indicated that additional use of TMZ could significantly reduce LVESD (31.00±4.33 mm from 33.29±2.11 mm).

### Serum level of cTnI

**Fig 3:**
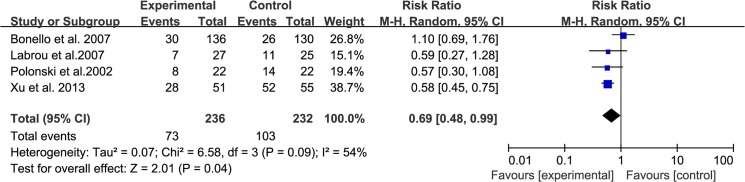
Forest Plots for Serum Level of cTnI.

A total of 4 trials (including 468 patients, 236 in the experimental group and 232 in the control group) analyzed the incidence of elevated cTnI >2 times the upper limit of normal, which was measured 24 hours after the PCI procedure. We used a random-effects model according to the test of heterogeneity (I^2^ = 54%, P = 0.09). The meta-analysis demonstrated a significant difference between the experimental and control groups (RR: 0.69, 95% CI: [0.48, 0.99], P = 0.04, Fig 3), indicating that addition of TMZ therapy was superior to the conventional treatment.

### Sensitivity analysis and meta-regression

Since significant heterogeneity across studies was observed for the incidence of elevated cTnI >2 times the upper limit of normal, we conducted a sensitivity analysis to assess the effect of each study on the pooled estimate under the random effects model [18, 19]. The detailed results are shown in Table 4. When one of the included studies was removed, I^2^ of the heterogeneity declined to 0%. The result still favored TMZ as a superior therapy. Meta-regression [20] indicated that the results did not vary according to age (beta: 0.29, 95% CI: [-0.07, 0.12], P = 0.55, S1 Fig), male gender (beta: 0.003, 95% CI: [-0.06, 0.03], P = 0.61, S2 Fig), or number of multivessel diseases (beta: 0.30, 95% CI: [-0.02, 0.01], P = 0.79, S3 Fig).

**Table 4:** Sensitivity Analysis of the Incidence of cTnI >2 Times the Upper Limit of Normal.

**Study omitted**	**RR (95% CI)**	***P* for heterogeneity**	***I*** ^***2***^
**Bonello et al. 2007**	0.58 (0.46, 0.73)	1	0%
**Labrou et al. 2007**	0.71 (0.45, 1.13)	0.05	70%
**Polonski et al. 2002**	0.73 (0.45, 1 18)	0.04	69%
**Xu et al. 2013**	0.77 (0.48, 1.23)	0.17	43%
**Study omitted**	**RR (95% CI)**	***P* for heterogeneity**	***I*** ^***2***^
**Bonello et al. 2007**	0.58 (0.46, 0.73)	1	0%
**Labrou et al. 2007**	0.71 (0.45, 1.13)	0.05	70%
**Polonski et al. 2002**	0.73 (0.45, 1 18)	0.04	69%
**Xu et al. 2013**	0.77 (0.48, 1.23)	0.17	43%

RR = relative risk; CI = confidence interval.

### Angina attacks during PCI

**Fig 4:**
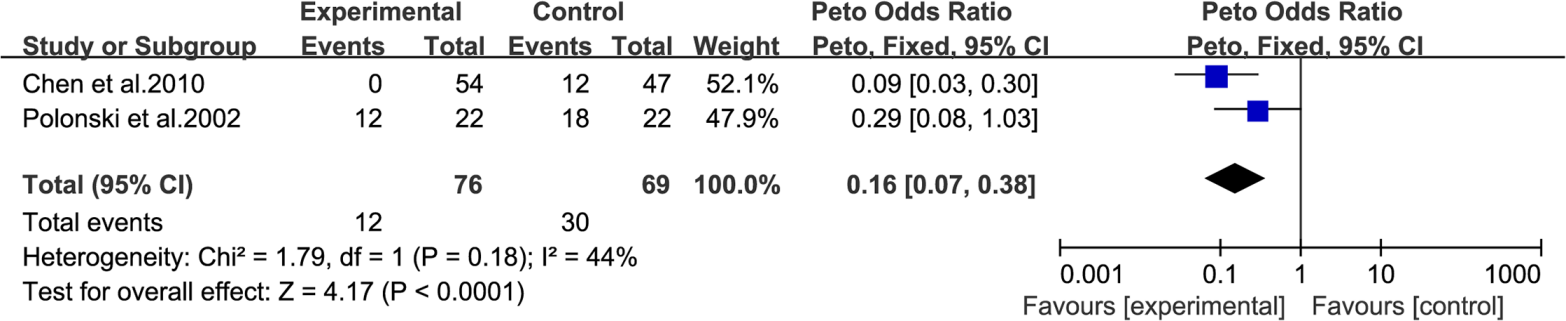
Forest Plots for Angina Attacks during PCI.

The occurrence of angina attacks during PCI, defined as angina symptoms reported during balloon inflation [13], was evaluated in 2 trials (including 145 patients: 76 in the experimental group and 69 in the control group). The data were analyzed using a fixed-effects model according to the test of heterogeneity (I^2^ = 44%, P = 0.18, Fig 4). The results demonstrated that the incidence of angina attacks in the experimental group was markedly lower than that in the control group (OR: 0.16, 95% CI: [0.07, 0.38], P<0.0001).

### Ischemic ST-T changes on the electrocardiogram during PCI

**Fig 5:**
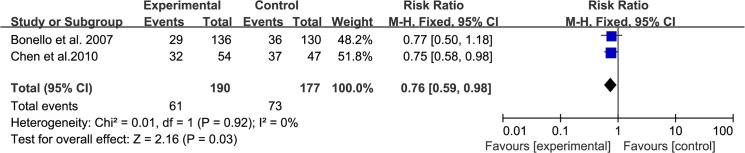
Forest Plots for Ischemic ST-T Changes on ECG during PCI.

The incidence of ischemic ST-T changes on the electrocardiogram (ECG) during PCI was assessed in 2 trials involving 367 patients (190 in the experimental group and 177 in the control group). We used a fixed-effects model according to the test of heterogeneity (I^2^ = 0%, P = 0.92). The meta-analysis showed that the incidence of ischemic ST-T changes on the ECG during PCI in the experimental group was significantly lower than that in the control group (RR: 0.76, 95% CI: [0.59, 0.98], P = 0.03, Fig 5).

### Serum level of BNP

**Fig 6:**
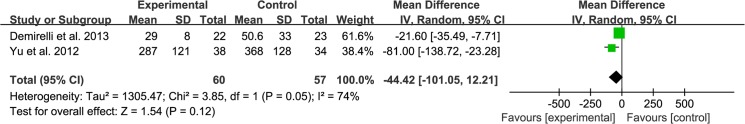
Forest Plots for Serum Level of BNP.

The serum BNP level was measured 30 days after PCI in 2 trials involving 117 patients (60 in the experimental group and 57 in the control group). Owing to the obvious heterogeneity between the 2 trials (I^2^ = 74%, P = 0.05), a random-effects model was used. After pooling the data, the experimental group achieved no significant reduction in serum level of BNP (WMD: -44.42, 95% CI: [-101.05, 12.21], P = 0.12, Fig 6).

## Discussion

With the rapid development of PCI over the past four decades, it has become one of the most important cardinal treatment options for coronary artery disease. The obvious advantages of this technique have reduced the incidence of major complications, including acute myocardial infarction, need for a coronary artery bypass graft, and death. In spite of these advantages, the rate of peri/post-procedural myocardial injury or necrosis, which may result in worse long-term outcome, has not substantially decreased [21, 22]. Therefore, we conducted the current meta-analysis to evaluate the effect of TMZ in the peri-procedural period of PCI. Our results indicated that additional use of TMZ was associated with considerable improvement of LVEF and reduction of serum cTnI level, angina attacks, and ischemic ST-T changes on the ECG during PCI. To the best of our knowledge, the present meta-analysis is the first to evaluate the effect of TMZ during PCI.

The cardioprotective effect of TMZ in the peri-procedural period of PCI was well established in this meta-analysis. A significant difference between the experimental group and control group was observed with regard to angina attacks and ischemic ST-T changes on the ECG during PCI. These observations indicate that TMZ could protect patients from myocardial injury. As for the sensitivity analysis of elevated cTnI level, when one of the included studies was excluded, the I^2^ of the heterogeneity declined to 0%. The analysis result still favored TMZ as a superior therapy over conventional therapy alone, and interestingly, the result of meta-regression favoring TMZ was consistent across different age, gender, and number of multivessel disease subsets. We also evaluated left ventricular function after follow-up. The follow-up duration in different trials ranged from 1 month to 12 months. The pooled results of these studies showed that TMZ therapy is associated with improvement in LVEF, and the sensitivity analysis still showed positive results that were similar to the comprehensive results. However, there was no significant difference in BNP between patients of the experimental and control groups.

The overall results indicated that additional use of TMZ therapy is superior to conventional drugs during PCI. Another meta-analysis that included six RCTs [23] also showed an additional beneficial effect of TMZ on patients with a coronary artery bypass graft, which included significantly decreased CK, CK-MB, troponin T, and troponin I, as compared to conventional treatment.

However, this meta-analysis could not provide powerful evidence for recommending TMZ in addition to conventional therapy for all patients undergoing PCI owing to the following limitations: First, only nine studies met our inclusion criteria and were included in this meta-analysis; furthermore, the sample size in six of the included studies was ≤100 patients. The Jadad score of all studies included in this meta-analysis was 3–5, indicating that the quality of these studies was not high. Second, we did not conduct a subgroup analysis of different types of patients (those with stable angina, unstable angina, and non-ST-segment elevation myocardial infarction), because most studies included patients with two or three types coronary artery disease. Third, the follow-up duration varied widely, from 1 month to 12 months. Last, the present study was a group-level meta-analysis because the data in this meta-analysis were available only at the level of the published reports, but we conducted a sensitivity analysis and meta-regression to avoid the potential bias, which showed consistent results with the overall results. These limitations might affect the statistical power for interpreting the results. Considering these limitations, studies with large samples and rigorous designs are needed in the future to enhance the power of the evidence for additional use of TMZ in patients undergoing PCI.

## Supporting Information

S1 PRISMA Checklist PRISMA Checklist. (PDF)

S1 Fig Meta-Regression of Age on Rates of Elevated cTnI >2 Times the Upper Limit of Normal (No Significant Interaction Was Noted). (TIF)

S2 Fig Meta-Regression of Male Gender on Rates of Elevated cTnI >2 Times the Upper Limit of Normal (No Significant Interaction Was Noted). (TIF)

S3 Fig Meta-Regression of Multivessel Disease on Rates of Elevated cTnI >2 Times the Upper Limit of Normal (No Significant Interaction Was Noted) (TIF)

S1 File PubMed Search Strategy. (PDF)

S2 File 35 Full-Text Articles Excluded. (PDF)
