# Supplementary figures and images for: Effect of Trimetazidine in Patients Undergoing Percutaneous Coronary Intervention: A Meta-Analysis
Source: PLoS One. 2015 Sep 14;10(9):e0137775. doi: 10.1371/journal.pone.0137775 (PMC4569304; doi:10.1371/journal.pone.0137775)

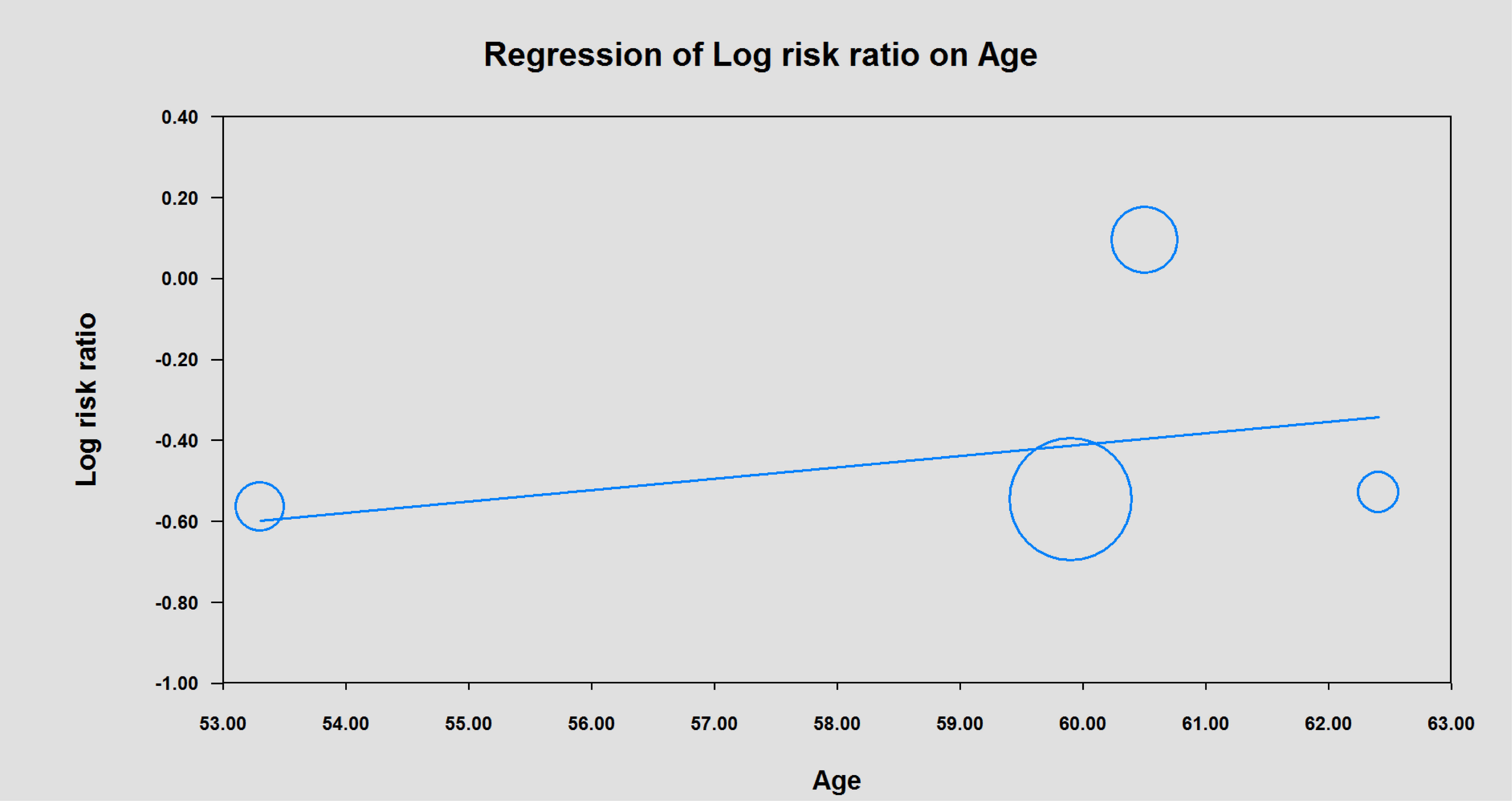

Supplement: S1 Fig — (TIF) [file pone.0137775.s002.tif]

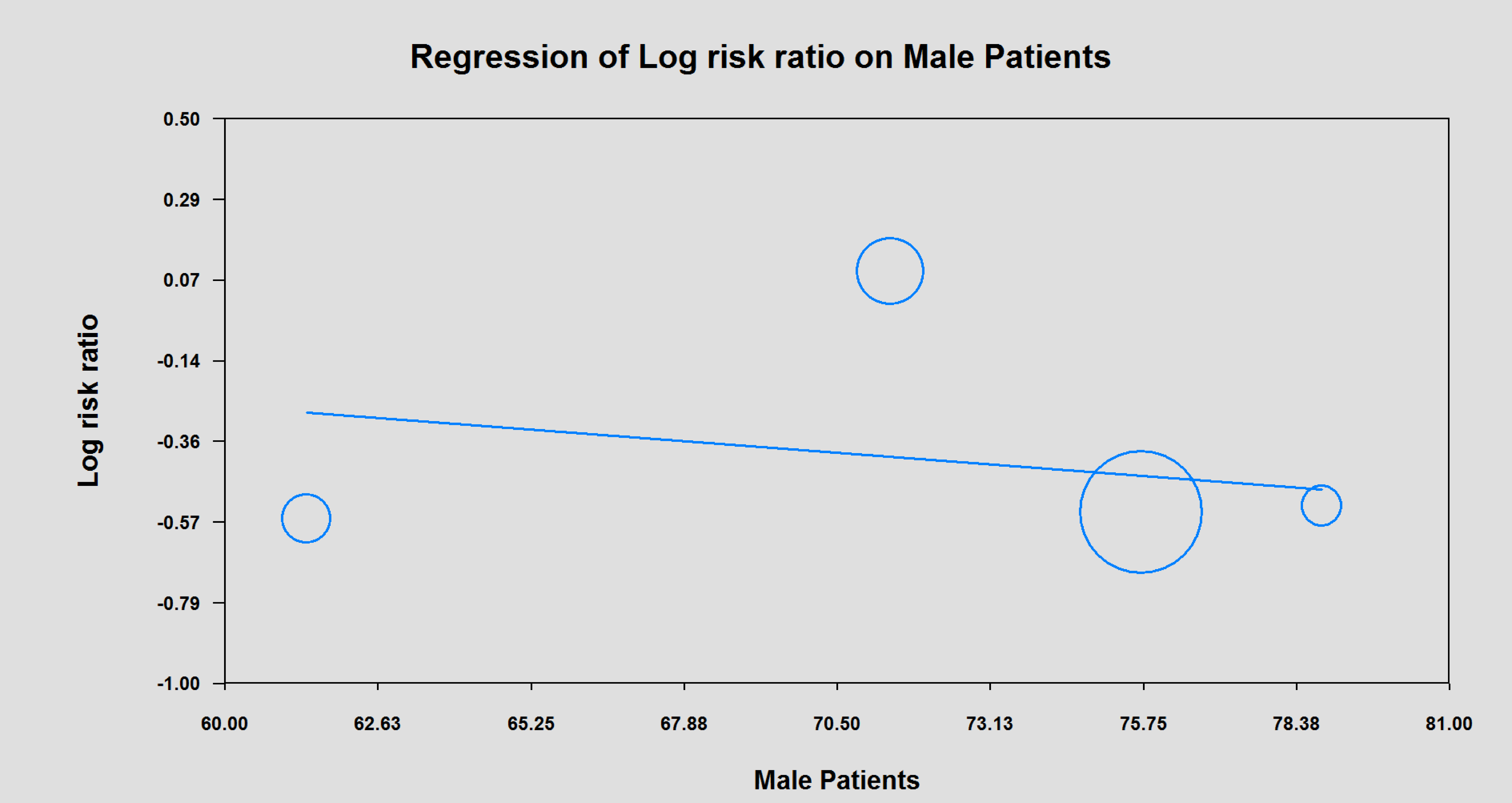

Supplement: S2 Fig — (TIF) [file pone.0137775.s003.tif]

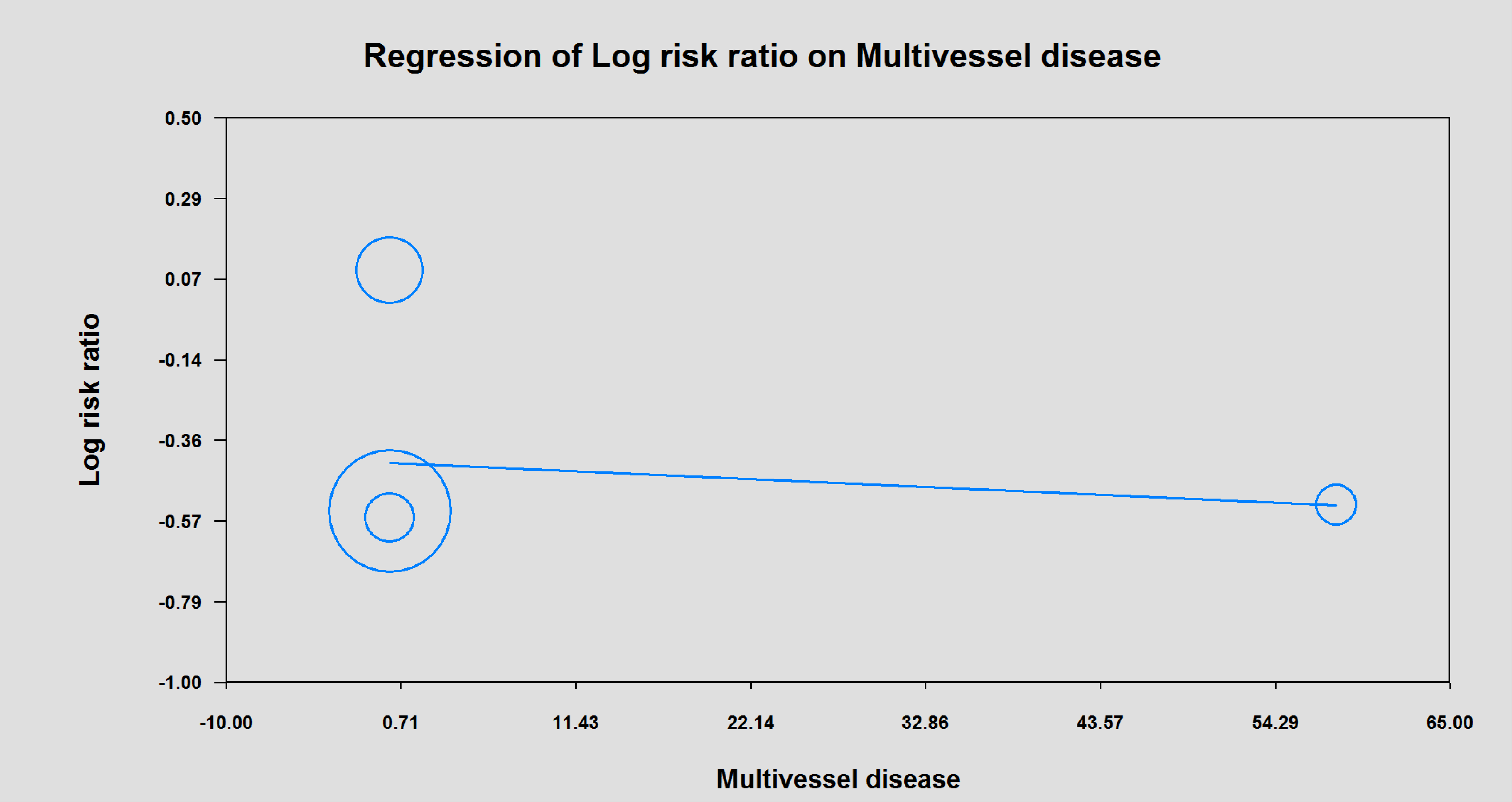

Supplement: S3 Fig — (TIF) [file pone.0137775.s004.tif]
