# Supplementary material for: Effect of Trimetazidine in Patients Undergoing Percutaneous Coronary Intervention: A Meta-Analysis
Source: PLoS One. 2015 Sep 14;10(9):e0137775. doi: 10.1371/journal.pone.0137775 (PMC4569304; doi:10.1371/journal.pone.0137775)
Supplement: S1 File — (PDF) [file pone.0137775.s005.pdf]

Search strategy in PubMed

#1 percutaneous coronary intervention

#2 percutaneous transluminal coronary angioplasty

#3 percutaneous transluminal angioplasty

#4 #1 or #2 or #3

#5 trimetazidine

#6 vastarel

#7 idaptan

#8 #5 or #6 or #7

#9 #4 and #8

Search details

((("percutaneous coronary intervention"[MeSH Terms] OR ("percutaneous"[All Fields] AND "coronary"[All Fields] AND "intervention"[All Fields]) OR "percutaneous coronary intervention"[All Fields]) OR ("angioplasty, balloon, coronary"[MeSH Terms] OR ("angioplasty"[All Fields] AND "balloon"[All Fields] AND "coronary"[All Fields]) OR "coronary balloon angioplasty"[All Fields] OR ("percutaneous"[All Fields] AND "transluminal"[All Fields] AND "coronary"[All Fields] AND "angioplasty"[All Fields]) OR "percutaneous transluminal coronary angioplasty"[All Fields])) OR ("angioplasty"[MeSH Terms] OR "angioplasty"[All Fields] OR ("percutaneous"[All Fields] AND "transluminal"[All Fields] AND "angioplasty"[All Fields]) OR "percutaneous transluminal angioplasty"[All Fields])) AND ((("trimetazidine"[MeSH Terms] OR "trimetazidine"[All Fields]) OR ("trimetazidine"[MeSH Terms] OR

"trimetazidine"[All Fields] OR "vastarel"[All Fields])) OR ("trimetazidine"[MeSH Terms] OR "trimetazidine"[All Fields] OR "idaptan"[All Fields]))
