# Supplementary material for: Effect of Trimetazidine in Patients Undergoing Percutaneous Coronary Intervention: A Meta-Analysis
Source: PLoS One. 2015 Sep 14;10(9):e0137775. doi: 10.1371/journal.pone.0137775 (PMC4569304; doi:10.1371/journal.pone.0137775)
Supplement: S2 File — (PDF) [file pone.0137775.s006.pdf]

1. Effect of trimetazidine on T-peak to T-end interval in patients with unstable angina pectoris after percutaneous coronary intervention. (In Chinese) Chinese Circulation Journal, 2014, 29: 776.

   The reason for exclusion: The outcomes do not meet our inclusion criteria.

2. Effects of trimetazidine on endothelial function of patients with multivessel disease. (In Chinese) Shandong Medical Journal, 2009, 49: 68-69.

   The reason for exclusion: The outcomes do not meet our inclusion criteria.

3. Effects of trimetazidine on serum levels of IL-6 and CRP of UAP patients undergoing percutaneous coronary intervention. (In Chinese) Journal of Clinical Cardiology (China), 2007, 23: 15-17.

   The reason for exclusion: The outcomes do not meet our inclusion criteria.

4. Protective effect of trimetazidine on contrast-induced in nephropathy in acute coronary intervention. (In Chinese) Chinese Journal of Coal Industry Medicine, 2014, 9: 003.

   The reason for exclusion: The outcomes do not meet our inclusion criteria.

5. Prevention of contrast-induced nephropathy with trimetazidine in patients with renal insufficiency. (In Chinese) Journal of Wenzhou Medical University, 2014, 44: 675-678.

   The reason for exclusion: The outcomes do not meet our inclusion criteria.

6. Effect of tongxinluo capsule combined with trimetazidine in hypersensitive C - reactive protein and blood lipid for patients with angina pectoris after PCI. (In Chinese) Practical Journal of Cardiac Cerebral Pneumal and Vascular Disease, 2012, 20: 1288-1290.

   The reason for exclusion: The outcomes do not meet our inclusion criteria.

7. Myocardial protection during percutaneous transluminal coronary angioplasty: effects of trimetazidine. European Heart Journal, 1992, 13:1109-1115.

   The reason for exclusion: The outcomes do not meet our inclusion criteria.

8. The role of trimetazidine in patients subjected to surgical revascularization. Archives of Medical Science, 2007, 3: S52.

   The reason for exclusion: It is not a RCT.

9. Effect of atorvastatin combined with trimetazidine on coronary heart disease. (In Chinese) Practical Journal of Cardiac Cerebral Pneumal and Vascular Disease, 2014, 22: 28-30.

   The reason for exclusion: It is not a RCT.

10. Effects of danhong injection combined with trimetazidine on postoperative myocardial injury and inflammatory factors after percutaneous coronary intervention in patients with acute myocardial infarction. (In Chinese) Chinese Journal of Rural Medicine and Pharmacy, 2015, 22: 48-49.

    The reason for exclusion: It is not a RCT.

11. Influence of trimetazidine on PCI-induced myocardial injury and postoperative recurrence of angina pectoris. (In Chinese) Chinese Journal of cardiovascular Rehabilitation Medicine, 2014, 23: 666-668.

    The reason for exclusion: It is not a RCT.

12. Clinical effect of trimetazidine on patients undergoing PCI. (In Chinese) Contemporary Medicine, 2012, 18: 153-154.

    The reason for exclusion: It is not a RCT.

13. Effect of trimetazidine to PCI on the reperfusion of acute myocardial infarction. (In Chinese) Chinese Journal of Practical Internal Medicine, 2009, 29: S1.

    The reason for exclusion: It is not a RCT.

14. Loading dose of trimetazidine in percutaneous coronary interventional therapy before the operation. (In Chinese) Guide of China Medicine, 2012, 10: 178-179.

    The reason for exclusion: It is not a RCT.

15. Effect of trimetazidine on the outcome and prognosis in aged patients with multivessel coronary artery disease undergoing incomplete revascularization by percutaneous coronary intervention. (In Chinese) Journal of Cardiovascular and Pulmonary Diseases, 2012, 31: 422-424.

    The reason for exclusion: It is not a RCT.

16. Effect of trimetazidine vs. tirofiban for patients of interventional therapy after myocardial infarction. (In Chinese) China Health Industry, 2013, 10: 134-134.

    The reason for exclusion: It is not a RCT.

17. Effects of trimetazidine in patients with acute myocardial infarction: data from the Korean Acute Myocardial Infarction Registry. Clinical Research in Cardiology, 2013; 102: 915-922.

    The reason for exclusion: It is not a RCT.

18. Myocardial protection during coronary artery bypass graft surgery: a randomized, double-blind, placebo-controlled study with trimetazidine. Anesthesia & Analgesia, 1996, 82: 712-718.

    The reason for exclusion: The intervention of patients is not appropriate. It is CABG instead of PCI.

19. Cardioprotective effects of atorvastatin plus trimetazidine in percutaneous coronary intervention. Pakistan journal of medical sciences, 2013, 29(2): 545.

    The reason for exclusion: Type of intervention does not meet our inclusion criteria.

20. Effect of trimetazidine in alliance with atovastatin therapy on myocardiac injury and inflammatory factors in unstable angina during perioperative period of percutaneous coronary intervention. (In Chinese) Chinese Journal of Interventional Cardiology, 2014, 11: 004.

    The reason for exclusion: Type of intervention does not meet our inclusion criteria.

21. Effects of compound danshen dripping pills and trimetazidine on restenosis after PCI of patients with myocardial infarction. (In Chinese) Chinese Community Doctors, 2011: 168-168.

    The reason for exclusion: Type of intervention does not meet our inclusion criteria.

22. Influence of nicorandil and trimetazidine on patients with recurrence of angina pectoris after percutaneous coronary intervention. (In Chinese) Occupation and Health, 2012, 5: 057.

    The reason for exclusion: Type of intervention does not meet our inclusion criteria.

23. Effects of trimetazidine combined with atorvastatin on postoperative patients with unstable angina pectoris. (In Chinese) Hebei Medical Journal, 2013, 35: 1173-1174.

    The reason for exclusion: Type of intervention does not meet our inclusion criteria.

24. Effects of trimetazidine and atorvastatin on postoperative patients with unstable

angina. (In Chinese) Fujian Medical Journal, 2010, 32: 140-141.

The reason for exclusion: Type of intervention does not meet our inclusion criteria.

25. Effects of trimetazidine and atorvastatin on postoperative patients with unstable angina. (In Chinese) Hebei Medical Journal, 2011, 33: 2612-2613.

The reason for exclusion: Type of intervention does not meet our inclusion criteria.

26. Effects of combined treatment with trimetazidine and atorvastatin on myocardial protection of patients undergoing percutaneous coronary intervention. (In Chinese) Pharmaceutical Care and Research, 2011, 11: 107-110.

The reason for exclusion: Type of intervention does not meet our inclusion criteria.

27. Influence of trimetazidine joint rosuvastatin on the prognosis of elderly multivessel coronary heart disease undergoing incomplete revascularization. (In Chinese) Journal of Community Medicine, 2014, 12.

The reason for exclusion: Type of intervention does not meet our inclusion criteria.

28. Trimetazidine combined compound danshen pill on the efficacy of interventional therapy of coronary heart disease. (In Chinese) Shaanxi Medical Journal, 2003, 32: 252-253.

The reason for exclusion: Type of intervention does not meet our inclusion criteria.

29. Comparative study for trimetazidine and nicorandil in patients with PCI related myocardial injury. (In Chinese) Chinese Circulation Journal, 2014, 29: 256-260.

The reason for exclusion: Type of intervention does not meet our inclusion criteria.

30. Observation of trimetazidine in treatment of senile coronary heart disease patients with three-vessel disease. (In Chinese) Practical Journal of Cardiac Cerebral Pneumal and Vascular Disease, 2012, 20: 1491-1492.

The reason for exclusion: Type of intervention does not meet our inclusion criteria.

31. Influence of rosuvastatin combined with trimetazidine on percutaneous coronary intervention- related myocardial injury. (In Chinese) Journal of New Medicine, 2014, 45: 249.

The reason for exclusion: Type of intervention does not meet our inclusion criteria.

32. Effects on serum lipid and cardiac function of tongxinluo combined with

trimetazidine in the treatment of patients with acute ST -elevation myocardial infarction after underwent percutaneous coronary intervention. (In Chinese) China Medical Herald, 2014, 11: 66-69.

The reason for exclusion: Type of intervention does not meet our inclusion criteria.

33. Trimetazidine may protect the myocardium during cardiac surgery. The Heart Surgery Forum 2009; 12: 175–179.

The reason for exclusion: The intervention of patients is not appropriate. It is CABG instead of PCI.

34. Is there any benefit of preoperative oral trimetazidine in coronary artery bypass graft? Journal of the Pakistan Medical Association 2012; 62: 1271-1276.

The reason for exclusion: The intervention of patients is not appropriate. It is CABG instead of PCI.

35. Cardioprotective effects of atorvastatin plus trimetazidine in percutaneous coronary intervention. Pakistan Journal of Medical Sciences 2013; 29: 545-548.

The reason for exclusion: Type of intervention does not meet our inclusion criteria.
